# Probing the local thermal expansion coefficient of single liquid Sn nanoparticles using EELS in STEM

**DOI:** 10.1038/s41598-025-88496-1

**Published:** 2025-02-13

**Authors:** A. Kryshtal, O. Khshanovska

**Affiliations:** Faculty of Metals Engineering and Industrial Computer Science, AGH University of Krakow, Al. Mickiewicza 30, Krakow, 30-059 Poland

**Keywords:** Nanoparticles, Thermal expansion coefficient, Sn, In situ TEM, EELS, Materials science, Nanoscience and technology

## Abstract

We investigated the local coefficient of thermal expansion (CTE) along the diameter of a 190 nm Sn nanoparticle supported on a thin Si₃N₄ substrate. The surface and volume plasmon energies of the liquid Sn nanoparticle were measured as a function of temperature using spatially resolved valence electron energy loss spectroscopy in a scanning transmission electron microscope. We found that the slope of the volume plasmon energy versus temperature gradually increased near the surface, revealing a thermal expansion gradient in liquid nanoparticles. This effect was confined to a 3 nm-thick surface layer, where the CTE was over 1.5 times larger than that of the nanoparticle core. Meanwhile, the CTE of the core was found to be smaller than the reference value for liquid Sn, which is attributed to mechanical constraints imposed by the thin Si₃N₄ substrate. We demonstrated that temperature-induced changes in surface plasmon energy provide insights into the CTE of the outmost surface layer of Sn nanoparticles.

**Supplementary Information:**

The online version contains supplementary material available at 10.1038/s41598-025-88496-1.

Metal nanoparticles exhibit unique properties that distinguish them from their bulk counterparts, such as quantum size effects, a high surface-to-volume ratio, and distinctive optical, electrical, and catalytic behaviors^1^. These properties have facilitated a wide range of applications across various fields, including medicine, electronics, energy, and environmental science. However, the properties of metal nanoparticles are not necessarily uniform along their diameter, and there is often a difference between the surface and the core of the nanoparticle. For instance, the melting of nanoparticles is not homogeneous; it starts at the surface at a temperature below the equilibrium melting point and the liquid overlayer grows and moves into a solid as the temperature increases^2,3^. The frequency of a collective oscillations of the free electron gas density at the surface and in the core of nanoparticles differs substantially. A strong scattering and absorption of electromagnetic waves at the surface result in numerous applications of nanoparticles in physics, biology, and medicine^4^. The electronic structure at the surface often differs from that of the bulk, playing an important role in enhanced catalytic activity in nanoscale metallic systems^5^.

The curvature of the surface has a pronounced effect on the properties of nanoparticles, particularly at sizes below approximately 20 nm. It is often supposed that Laplace pressure compresses nanoparticles. The degree of compression depends on the surface tension/stress and the size of the nanoparticle^6^. The concept of Laplace pressure was used to interpret the size-dependent depression in the lattice constant^7,8^and melting point^9,10^of metal nanoparticles, as well as enhanced sintering^11,12^, increased chemical reactivity^13,14^ and other phenomena.

The direct measurement of the local properties in nanoparticles is a nontrivial task due to their small size and the limited spatial resolution of most characterization techniques. Nevertheless, recent developments in TEM instrumentation and data analysis have enabled investigations of local properties in nanomaterials at unprecedented levels. Thus, electron energy loss spectroscopy (EELS) combined with scanning transmission electron microscopy enabled the investigation of the thermal expansion coefficient (CTE) of nanoparticles^15,16^, grain boundaries^17^, and even mapping of CTE in freestanding 2D materials^18^. The value of thermal expansion can be deduced from temperature-induced plasmon peak shift. On the contrary, temperature dependence of the volume plasmon energy can be used for local temperature determination resulting in the emergence of a plasmon nanothermometry technique^19^.

At the same time, mapping of CTE in liquid nanoparticles has not been performed yet, to our best knowledge. It has been well established that liquid metals follow a simple relationship between their volumetric expansivities and melting temperatures *T*_*m*_^20^, i.e.

1 $$\:{\alpha\:}_{V}=\frac{0.09}{{T}_{m}}$$

Since metal nanoparticles exhibit a surface premelting effect, the CTE of their surface is expected to be higher than that of their core. To address this, we studied the local thermal expansion coefficient along the diameter of liquid Sn nanoparticles using the spatially resolved valence EELS in STEM.

## Results

**Fig. 1:**
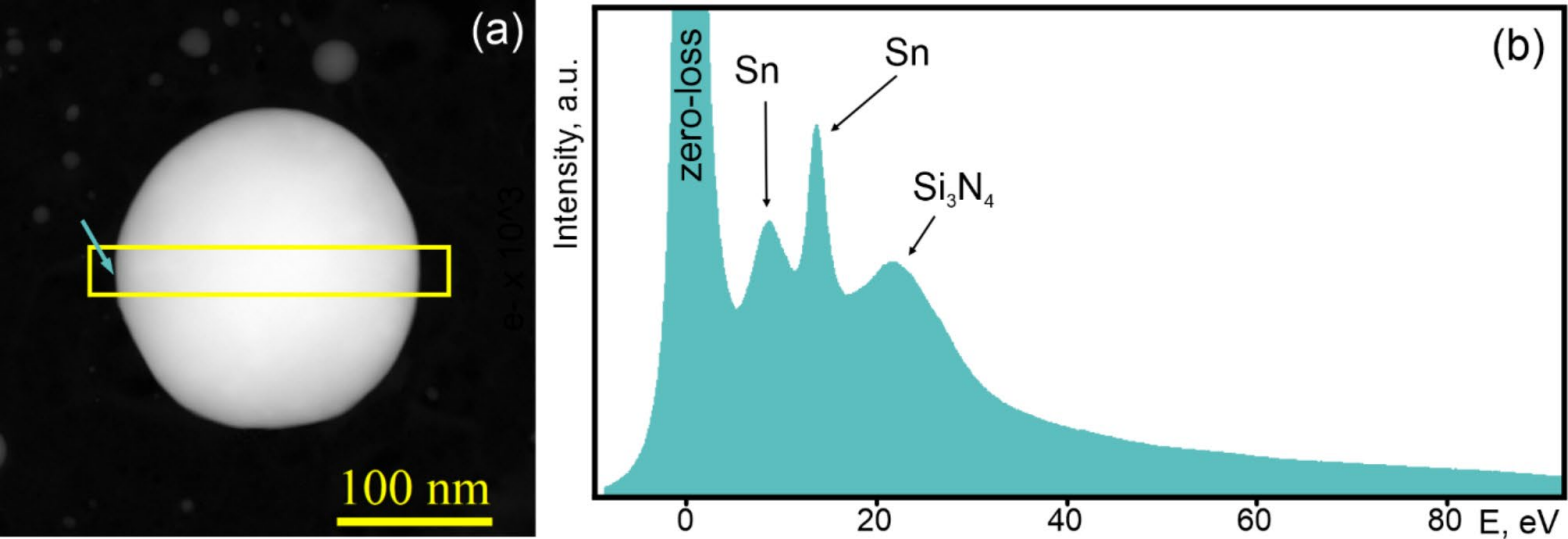
(**a**) HAADF STEM image of a Sn nanoparticle on a Si_3_N_4_ substrate at room temperature, and (**b**) EEL spectrum from its surface region (indicated by the arrow in (**a**)). The yellow rectangle in (**a**) marks the area where the EEL spectral image was acquired.

Several liquid Sn nanoparticles, ranging in size from 315 nm to 41 nm, were studied. Figure 1a shows a representative particle for which the data analysis was the most accurate and complete. It has a spherical shape with a diameter of about 190 nm. Nanoparticles of this size are not subject to size-related effects and are resistant to beam-induced damage, enabling robust EELS signals for both volume and surface plasmon resonances. The nanoparticle was cooled from 750 °C to 235 °C in 50 °C steps, and a low-loss EEL spectrum map was acquired from the same region of the Sn nanoparticle (marked by a rectangle in Fig. 1a) at each temperature. Figure 1b shows an integrated EEL spectrum from the Sn surface and substrate, capturing all characteristic energy losses in a single spectrum. Three well-separated energy loss peaks are seen in Fig. 1b along with the zero-loss peak. The peak at ≈22 eV corresponds to a plasmon resonance of Si_3_N_4_ support film (*E*_*SiN*_), and the peaks at ≈13 eV and ≈9 eV correspond to the volume (*E*_*p*_) and surface (*E*_*s*_) excitations of Sn, respectively. The relative intensity of the peaks varied along the diameter of the nanoparticle. The volume plasmon peak was dominant in the core but decayed at the surface due to the excitation of surface plasmons (the so-called Begrenzungs effect), which became the dominant peak at the particle-vacuum interface. Since the thickness of the nanoparticle varied along its radius, the energy of the volume plasmon peak at the core and near the surface had different values likely due to enhanced surface interactions^21^. Therefore, measurements of the temperature-induced shift of the plasmon peaks were only possible in the same region of the nanoparticle.

The thickness of the substrate was determined from its EELS spectrum using the log-ratio method. The relative thickness t/λ was ≈0.1, which corresponded to a Si_3_N_4_ substrate thickness of ≈14 nm. The contact angle θ of the Sn nanoparticle with the substrate (assuming it is flat and rigid) was assessed from its height *H* and radius *R* using the equation:

2 $$\:\theta\:={arccos}\left(1-\frac{H}{R}\right)$$

The height of the nanoparticles *H *was determined using the relative thickness (t/λ) value at the center of the nanoparticle (reduced by the substrate thickness t/λ = 0.1) and the mean free path of electrons for Sn (λ = 116.5 nm)^22^. The radius of the nanoparticle *R *was measured directly from the HAADF STEM images. The assessments showed that the contact angle gradually increased during cooling, from ≈116° at 750 °C to ≈125° at 235 °C. This trend is expected, as the surface tension of liquids decreases with increasing temperature^23^.

**Fig. 2:**
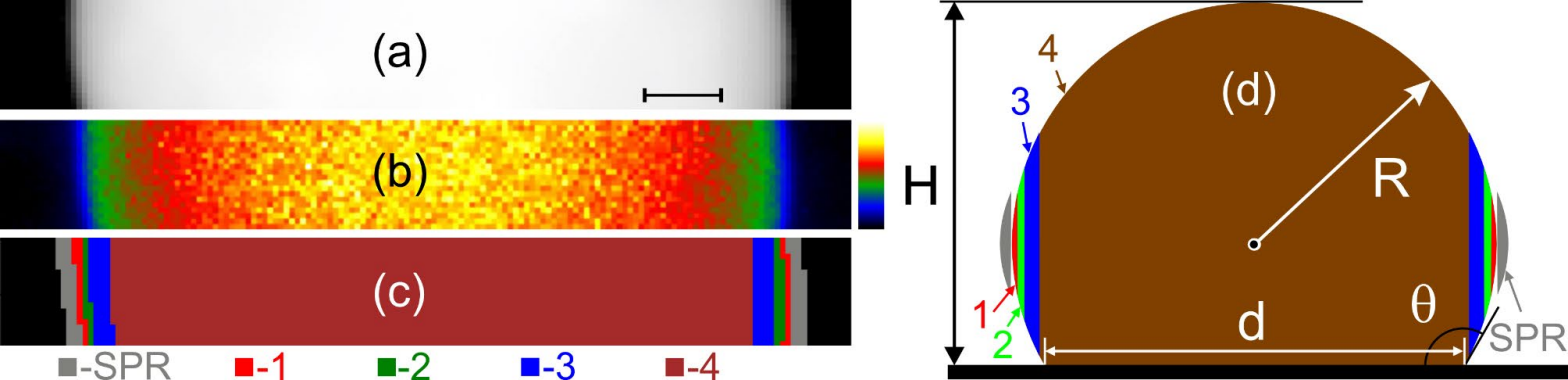
(**a**) HAADF STEM image of a selected part of the liquid Sn nanoparticle (yellow rectangle in Fig. 1a) at 700 °C, (**b**) false color EELS relative thickness map, and (**c**) false color map showing the regions “1”-“4” and “SPR” that were analyzed. (**d**) Schematic side-view of the Sn nanoparticle, indicating the approximate location of the analyzed regions. *R* and *H* represent the radius and height of the particle, respectively, and *d* represents the diameter of the contact area with the substrate. The scale bar is 20 nm in (a), and 0.1–1.6 t/λ in (b).

**Fig. 3:**
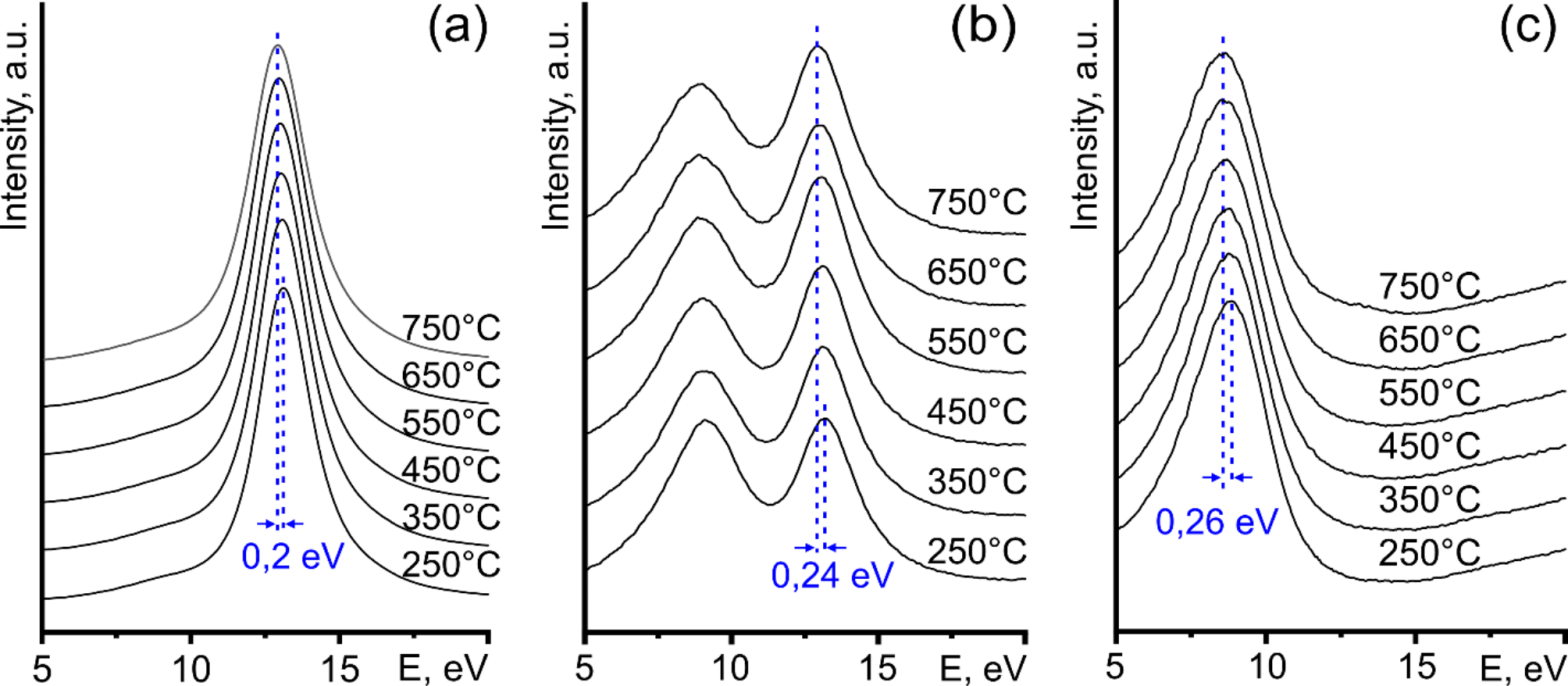
Raw EEL spectra of plasmon peaks from (**a**) the core (area 4 in Fig. 2c), (**b**) the surface (area “2” in Fig. 2c), and (**c**) the particle-vacuum interface (area “SPR” in Fig. 2c) of the liquid Sn nanoparticle at several temperatures between 250 °C and 750 °C. Vertical dashed lines indicate the plasmon peak centers for 250 °C and 750 °C, along with the corresponding temperature shift.

The image of the region of the liquid Sn nanoparticle used for EELS analysis is shown in Fig. 2a, along with its false-color relative thickness map (Fig. 2b). The region of interest was sampled based on the t/l value into four areas (“1”–“4”) along the diameter of the nanoparticle, with an additional area labeled “SPR” located near the particle-vacuum interface (Fig. 2c). A schematic side view of the Sn nanoparticle on a flat substrate with the wetting angle of 120° showing the approximate locations of the areas under study is presented in Fig. 2d. Area “SPR”, which is outside the nanoparticles, was used for studying the surface plasmon resonance in Sn. Areas “1” and “2” represent the surface and near-surface layers, respectively, and are each 1 pixel or 1.5 nm in width. Areas “3” and “4”, shown in blue and brown colors in Fig. 2c, refer to the volume of the nanoparticle. The Si_3_N_4_ substrate is shown in black color in Fig. 2c. The integrated EEL spectra from core (“4”), near-surface (“2”) areas, and particle-vacuum interface (“SPR”) at different temperatures are presented in Fig. 3a, b, and c, respectively. Note that the peaks maintain the symmetry of their shape across the entire temperature range, revealing the consistency of the measurements. The full width at half maximum (FWHM) of the volume plasmon peak (Fig. 3a) gradually increased from 2.14 eV at 235 °C to 2.4 eV at 750 °C. The FWHM of the surface plasmon peak (Fig. 3c) varied between 3.48 eV and 3.7 eV in the same temperature range. At the same time, the centers of both bulk and surface plasmon peaks shifted toward higher energies during cooling, while maintaining their symmetrical shape (Fig. 3).

**Fig. 4:**
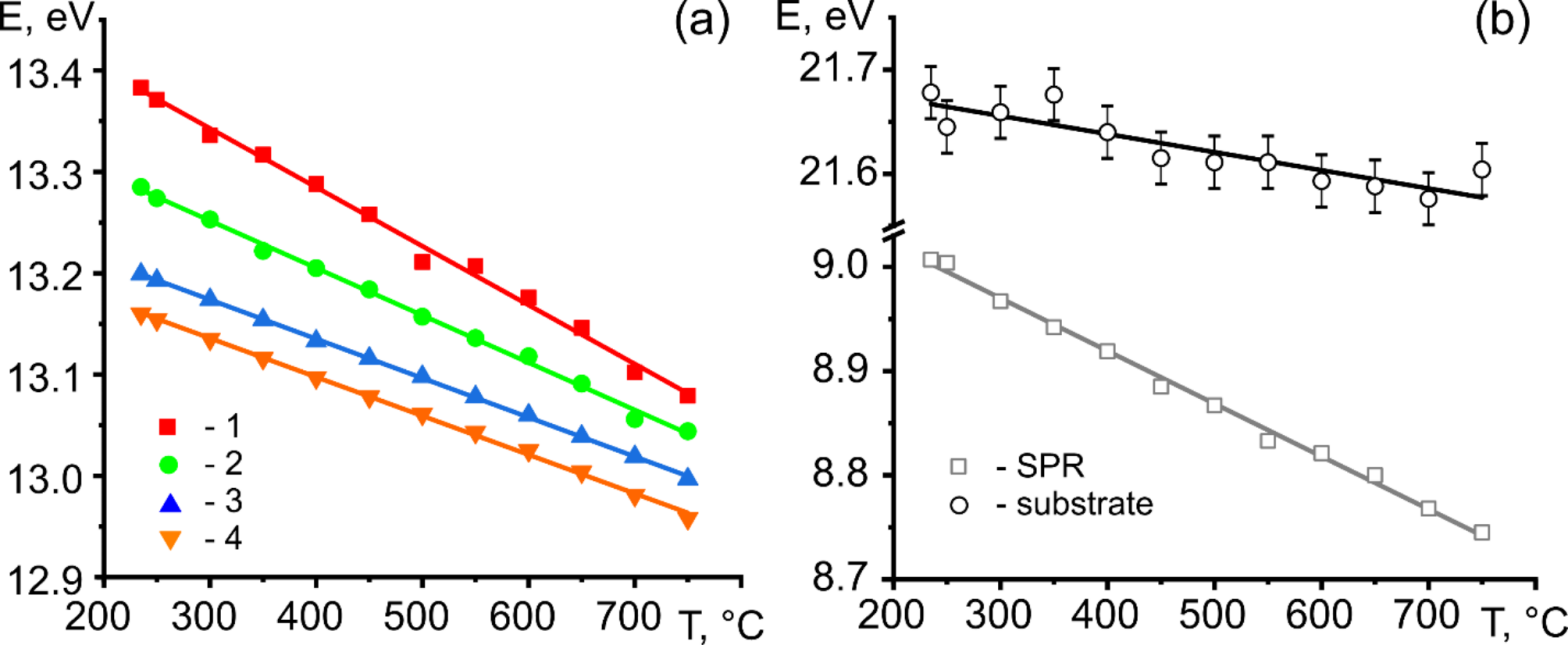
(**a**) Variation in *E*_*p*_ for regions “1”-“4” of liquid Sn nanoparticle as a function of temperature. (**b**) The change of *E*_*s*_ of the Sn nanoparticle and *E*_*p*_ of Si_3_N_4_ substrate versus temperature. The solid lines represent a linear fit.

The variation in volume plasmon energy *E*_*p*_ for areas “1”-“4”, substrate *E*_*SiN*_, and surface plasmon excitation *E*_*s*_ ( “SPR”) as a function of temperature is shown in Fig. 4a, b. A linear relationship between the plasmon energy and temperature is evident for each dataset. The determined values of d*E/*d*T* are summarized in Table 1. The rate at which *E*_*p*_ decreases with increasing temperature varies along the radius of the nanoparticle. The lowest rate of −0.383 meV/°C had the core, and the largest rate of −0.58 meV/°C was observed for the surface layer of the Sn nanoparticle.

It is well known that the volume plasmon energy *E*_*p*_is specific to the material and within Drude model is written as^24^

3 $$\:{E}_{p}\left(\text{T}\right)={\hbar}\sqrt{\frac{n\left(T\right){e}^{2}}{m{\epsilon\:}_{0}}}$$

where ħ – is the reduced Planck constant, *n* – is the density of valence electrons, *e* and *m* are the electron charge and mass, respectively, *ε*_0_ – is the vacuum dielectric constant. The electron density is given by the expression $$\:n\left(T\right)=\raisebox{1ex}{$N$}\!\left/\:\!\raisebox{-1ex}{$V\left(T\right)$}\right.$$, where N is the total number of free electrons and V – is the volume. Consequently, the temperature shift of the volume plasmon energy d*E*_*p*_*/*d*T* is associated with the volume change d*V/*d*T*. It is more convenient to express the volume change in terms of thermal expansion coefficient $$\:{\alpha\:}_{V}=\frac{dV}{dT}\frac{1}{V}$$. The latter is related to *E*_*p*_ change as

4 $$\:{\alpha\:}_{V}=-\frac{2}{{E}_{p}\left(T\right)}\frac{d{E}_{p}}{dT}$$

Therefore, the rate of the *E*_*p*_change is the measure of the volumetric expansion coefficient of the material. Expression (4) and its derivatives were widely used for the determination of CTE in solids^16–18,25^and less in liquids^26^, although liquids exhibit similar behavior to solids under the free electron model^27^. The mechanism for thermal expansion of molten metals is different from that of crystalline materials and is primarily due to an increase in the free volume of holes^28,29^. The Drude model (Eq. 3) does not explicitly consider vacancies and holes, however, the voids increase the total volume *V*, leading to a reduction in the mean electron density of liquids.

**Fig. 5:**
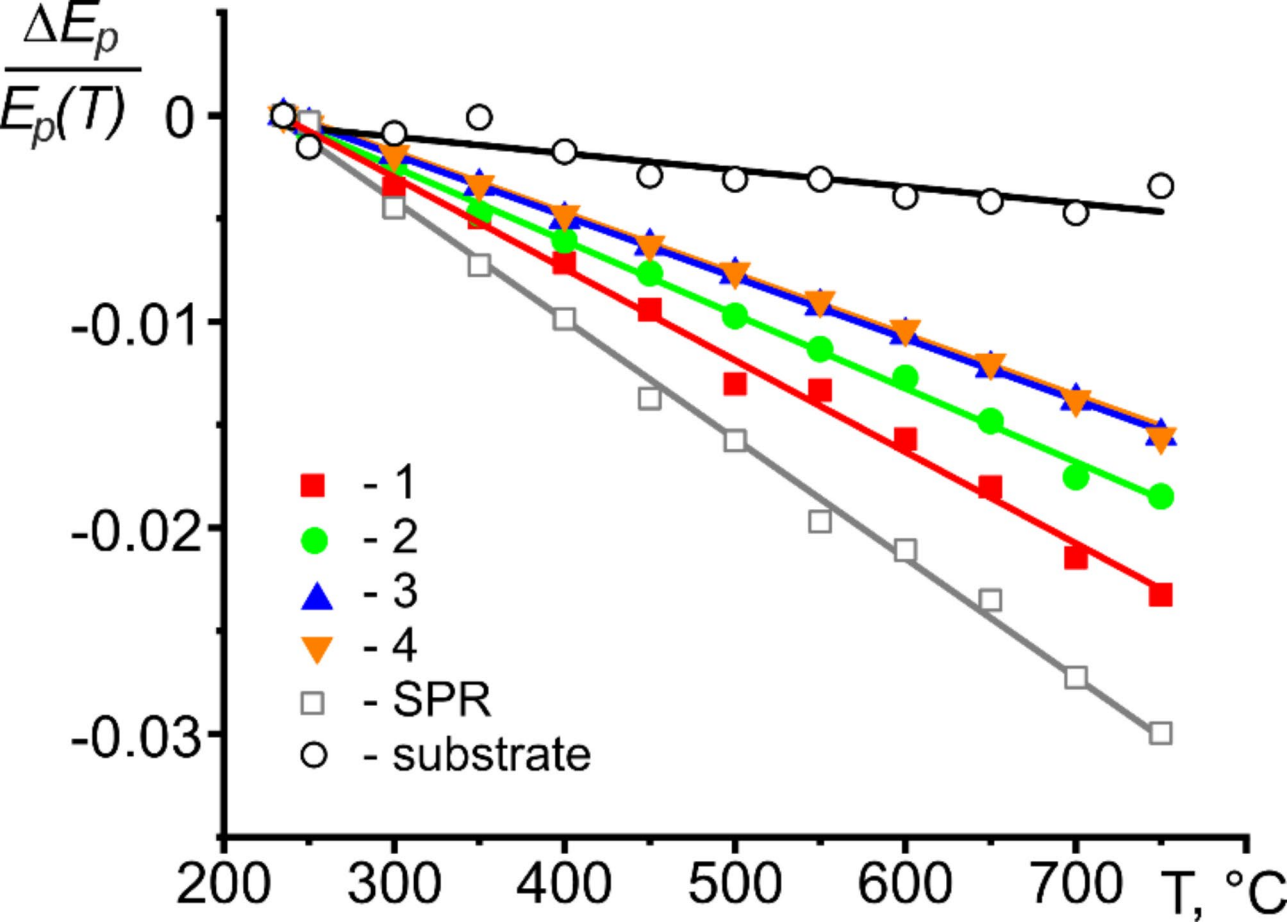
The relative change of the plasmon peak center versus temperature. Open squares – are for surface plasmon resonance of liquid Sn, open circles - are for Si_3_N_4_ substrate, and closed symbols - are for the volume plasmon resonance from regions “1”-“4” of the Sn nanoparticle. The solid lines represent a linear fit.

Figure 5 shows the relative change of the plasmon peak center versus temperature for the data presented in Fig. 4a and b. The slope of the straight lines gives α_*V*_/2. Note that no temperature variation in α_*V*_ is visible in the 235 –750 °C temperature range. The determined local volumetric thermal expansion coefficient α_*V*_ along the radius of the 190 nm-size Sn nanoparticle is presented in Table 1, along with the thermal expansion coefficient of the substrate. The results revealed a CTE gradient along the radius of the nanoparticle. The measurement results for nanoparticles with sizes of 315 nm, 53 nm, and 41 nm are available in the Supplementary Material. They support and validate the conclusions drawn for the 190 nm-sized particle.

Surface plasmons, which are longitudinal waves of charge density that travel along a surface, in the simplest case of a vacuum/metal interface, have the energy $\:{E}_{s}={E}_{p}/\sqrt{2}$^30^. Hence, the temperature-induced shift of the surface plasmon resonance energy provides information on the thermal expansion of the surface of the Sn nanoparticle, as shown in Fig. 5. The α_*V*_ value derived from the shift in volume plasmon peak energy converged with the α_*V*_ value obtained from the change in surface plasmon peak energy at the surface.

**Table 1:** Temperature shift of the plasmon energy d*E*/d*T* and local coefficient of volumetric thermal expansion α_*V*_ of liquid Sn nanoparticle and Si_3_N_4_ substrate in the temperature range of 235–750 °C. D – is the approximate inward distance from the surface of the Sn nanoparticle. The error in d*E*/d*T* represents the standard error of the slope.

Area	d, nm	dE/dT, [meV/°C]	α_V_, 10^−5^[°C^−1^]
Substrate	-	−0.17±0.03	1.5
SPR	-	−0.51±0.01	12
1	0–1.5	−0.58±0.01	8.9
2	1.5–3	−0.467±0.007	7.3
3	3–9	−0.387±0.002	5.96
4	> 9	−0.383±0.005	5.86

## Discussion

Two principal observations of our work should be discussed. Firstly, the measured thermal expansion coefficient of 5.86 × 10^−5^ °C^−1^ for the core of a 190 nm liquid Sn nanoparticle is substantially smaller than the value reported for liquid, which ranges from 9 × 10^−5^ °C^−1^ to 10 × 10^−5^ °C^−1^.^9,26,28,31^ It is also lower than the CTE of solid Sn, which is approximately 7.3 × 10^−5^ °C^−1^.^23^ This discrepancy cannot be attributed to the accuracy of the measurements; rather, it is attributed to mechanical constraints imposed by the substrate. Indeed, the Sn nanoparticle is not free but is adhered to the Si_3_N_4_ substrate (Fig. 2d). The size of the wetting area *d* (Fig. 2d), at a contact angle θ = 120°, reaches approximately 0.87 times the nanoparticle’s diameter. This ensures good thermal contact with the substrate. On the other hand, the Si_3_N_4_ substrate, with a CTE approximately six times lower than that of Sn (Table 1), significantly restricts the thermal expansion of the contact region of the Sn nanoparticle. The thermal expansion mismatch is partially relieved through out-of-plane deformation in 14-nm thick Si_3_N_4_substrate. Furthermore, the “soft” substrate substantially alters the wetting behavior in particle-substrate systems^32^. For a flat and rigid substrate, the wetting angle of a sessile droplet is described by the Young Eq. 3^33^

5 $$\:{\sigma\:}_{lv}cos\theta\:={\sigma\:}_{sv}-{\sigma\:}_{sl},$$

where *σ*_*lv*_, *σ*_*sv*_, and *σ*_*sl*_, are the liquid-vapor, solid-vapor, and solid-liquid interfacial tensions, respectively. This equation accounts for the in-plane balance of the interfacial tensions at the triple junction. However, the vertical component of *σ*_*lv*_ remains unbalanced, which on soft substrates, leads to the formation of a wetting ridge near the contact line (details are provided in the Supplementary Material). This ridge further constrains the thermal expansion of Sn nanoparticles by increasing the contact area between the particles and the substrate.

The electron microscopy images and EEL spectral images are 2D projections of a 3D sample. For instance, the EELS spectra from the volume of the nanoparticle (region 4 in Fig. 4a) represent a superposition of contributions from the surface, bulk, liquid/solid interface, and the Si₃N₄ substrate. Consequently, the measured CTE value in this region represents a mean value that lies between the CTE of Sn and Si₃N₄. We anticipate that the substrate’s stiffness and thickness, the degree of particle-substrate interaction, and the nanoparticle size will influence the measured coefficient of thermal expansion.

The second principal observation of the work is revealing the local thermal expansion gradient in single liquid Sn nanoparticles. We demonstrated that the local thermal expansion coefficient gradually increased towards the surface of the Sn nanoparticle. The effect was most pronounced within a 3 nm-thick surface layer, where its magnitude reached 50–100%. Consequently, the CTE in region “1” of the nanoparticle (Table 1) approached the reference value for Sn. The measured shift dE/dT in this region matches ultra-high vacuum studies of sub-surface volume plasmons in bead-shaped liquid Sn, conducted using high-resolution EELS^26^, confirming the consistency of our measurements.

The thermal expansion coefficient of the surface was 1.5–2 times greater than that of the core of Sn and approximately 20% greater than the reference value for liquid Sn. Notably, the observed CTE gradient was smooth, with the surface exhibiting the highest CTE value. Hence, we conclude that the Laplace pressure had a marginal effect on the thermal expansion of the 190 nm Sn nanoparticle.

In a homogeneous and isotropic liquid, several factors could induce increased CTE values at the free surface. These factors include a temperature gradient from non-uniform heating or cooling, surface and size effects, and material inhomogeneity (e.g., due to surface oxidation). Thermal radiation emission according to Planck’s law can create a small temperature gradient within the nanoparticle, leading to a higher temperature at the core compared to the surface. In this scenario, EELS analysis would yield a lower CTE value for the surface compared to near-surface regions; however, this was not the case in our study. We found no inhomogeneity in the HAADF image contrast of liquid Sn nanoparticles. The clear and strong surface plasmon excitation peaks in the EEL spectra (Fig. 3c), with energy characteristics of pure Sn, revealed an oxide-free surface of the nanoparticle over a temperature range of 250–750 °C.

Thus, we are confident that the observed increase in CTE is associated with the properties of the surface of the liquid Sn nanoparticle and can be understood in terms of atomic vibrations^20,34^. Surface atoms have fewer neighboring atoms compared to bulk atoms. Thus, a coordination number ratio of surface to bulk of about 0.713 was reported for the majority of liquid metals^34^. Since surface atoms are less constrained by bonding forces, they vibrate with greater amplitude. The enhanced vibrations increase the local thermal expansion at the surface relative to the bulk. As the amplitude of atomic vibrations increases, the likelihood of forming larger and more numerous voids at the surface also increases, resulting in a reduced local density of the nanoparticle. This aligns with previously published data on the thermal expansion of nanoparticles and grain boundaries. A fivefold increase of the volumetric thermal coefficient as the size of the Ag nanoparticles decreases from 30 nm to 11 nm was reported in Ref^35^. Additionally, about 1.6 times higher volumetric CTE was found in the Σ5 grain boundary of SrTiO_3_over a temperature range of 100 to 700 °C^17^.

In summary, the surface and volume plasmon energy of single Sn nanoparticles, as well as the volume plasmon energy of a 14-nm thick Si_3_N_4_ substrate, were studied over a temperature range of 250–750 °C. The analysis revealed a gradual increase in the slope of volume plasmon energy as a function of temperature near the surface, indicating the presence of a thermal expansion gradient in individual Sn nanoparticles.

The CTE of the Sn core was found to be smaller than the reference value for liquid Sn, which was attributed to the mechanical constraints imposed by the substrate. In contrast, the CTE of the surface was greater (1.5–2 times for the 190 nm nanoparticle) than that of the Sn core. This effect was most pronounced within a 3 nm-thick surface layer. Our results demonstrate that temperature-induced shifts in surface plasmon resonance energy provide insights into the CTE of the surface of Sn nanoparticles. The efficiency of in situ valence EELS in probing the local electronic structure of liquid nanoparticles was also demonstrated.

## Materials and methods

Two identical specimens with Sn nanoparticles on an amorphous silicon nitride (Si_3_N_4_) substrate were prepared as follows. First, a 40 nm-thick Sn thin film was deposited onto a room-temperature Si_3_N_4_ substrate of a nanochip (Wildfire_HB_ GT, DENSsolutions) using a DC magnetron sputtering system Prims 032 from PREVAC (Poland) under a vacuum of 7 × 10^−7^ Torr. The sample was then transferred to a transmission electron microscope (TEM) and annealed at a temperature of 750 °C. During annealing, the holder was retracted from the pole piece of the microscope for safety reasons. The high-temperature annealing process induced melting and dewetting of the Sn film, resulting in the formation of an island film with well-separated particles. Additionally, the high-temperature treatment ensured an oxide-free surface on the Sn nanoparticles^3^.

The first nanochip was used to study the thermal stability of liquid Sn nanoparticles in the TEM over a temperature range of 600–900 °C (details are provided in the Supplementary Material). The second nanochip was utilized to investigate the thermal expansion of individual nanoparticles as described below.

A probe Cs-corrected Titan Cubed G2 60–300 (FEI) electron microscope operated in STEM mode at an acceleration voltage of 200 kV and 80 kV was used for the in situ TEM studies. The microscope was equipped with a double tilt MEMS-based heating holder Wildfire D6 from DENSsolutions, a Gatan Image Filter (model 966), and Gatan Digiscan™ to produce spectral maps from electron energy loss spectroscopy data.

The in-situ experiments covered the temperature range of 235–750 °C. Measurements were made during a cooling cycle, with the temperature manually adjusted in 50 °C steps. The temperature readout of the heating holder was calibrated using the melting temperature of 232 °C for ≈ 200 nm-sized Sn nanoparticles, ensuring a temperature error of less than 2% within the operational range.

Images were acquired with a high-angle annular dark-field (HAADF) detector (collection angle of 47–200 mrad). Low-loss EEL spectrum images, 156 × 20 pixels in size, were acquired with a pixel size of 1.5 nm, a dwell time of 50 ms, and a beam current of approximately 40 pA. The total acquisition time was 2 min 49 s, resulting in an electron dose of 7 × 10^6^ e/nm^2^ per EELS SI. The EELS dispersion of 0.05 eV per channel, convergence, and collection semi-angles of 12 and 28 mrad, respectively, were used. No drift correction was applied. The energy spread of the electrons was approximately 1 eV, as measured from the full-width at half-maximum (FWHM) of the zero-loss peak.

The EEL spectral maps were analyzed with a dedicated Python code that utilized the open-source HyperSpy library^36^, providing fully automated analysis. First, a relative thickness t/λ (t – is the local thickness, λ - is the mean free path of electrons) map was produced using the log-ratio method^30^. The data were then divided by t/λ value into six sets, with each set corresponding to a specific region of the nanoparticle. For instance, for a ≈190 nm-sized nanoparticle, we used the following t/λ ranges for datasets 1–6: 0–0.15, 0.15–0.25, 0.25–0.35, 0.35–0.45, 0.45–0.6, and > 0.6. The EEL spectra in each dataset were summed into one high-quality spectrum. In this manner, EEL spectra from the substrate, surface, near-surface, and core regions of Sn nanoparticles were obtained. The approach accommodated temperature-induced shape fluctuations and specimen drift during data acquisition. Finally, the energy of the plasmon peak was determined for each region as a function of temperature. We used the Drude plasmon model^30^ for fitting Sn and Si_3_N_4_ plasmon peaks, and the Gaussian model for fitting the zero-loss peak (ZLP). The energy ranges of 6–11 eV and 10–16 eV were used for fitting the surface and volume plasmon peaks, respectively. For spectra exhibiting two plasmon peaks, the energy range of 6–16 eV and two fitting functions were applied. Because the electron beam with a relatively large energy spread was used in the study, limiting the accuracy of peak broadening analysis, elastic scattering and plasmon coupling effects were not considered in the peak analysis.

The model-based fitting of ZLP and plasmon peaks significantly improved the precision of the measurements, which is one of the main limitations of the EELS technique. The precision of the volume plasmon energy of Sn was evaluated as the difference between the maximum and minimum values in a dataset of 20 measurements taken from the central part of the nanoparticle, amounting to 0.005 eV (Fig. S4 in the Supplementary Material). The precision of the surface plasmon resonance of Sn and Si_3_N_4_ substrate volume plasmon peak energies was lower, at ±0.01 eV and ±0.025 eV, respectively.

It should be noted that the unavoidable delocalization of low energy loss signals practically limits the spatial resolution of the EELS technique. The delocalization width can be approximated as 17 nm/E^3/4^, where E is in eV^30^, and for energy losses of 13 eV it amounts to ≈ 2.5 nm.

## Electronic supplementary material

Below is the link to the electronic supplementary material.

Supplementary Material 1. Thermal stability of island Sn films on Si_3_N_4_ substrate. Deformation of the substrate under Sn nanoparticles. Figure S4. The measured volume plasmon Sn in a set of repeated EELS measurements. Results of measurements for other Sn nanoparticles. (PDF)

## Data Availability

The datasets generated during the current study are available from the corresponding author on reasonable request.
