# Publisher Correction: Probing the local thermal expansion coefficient of single liquid Sn nanoparticles using EELS in STEM

**DOI:** 10.1038/s41598-025-94236-2

**Published:** 2025-04-03

**Authors:** A. Kryshtal, O. Khshanovska

**Affiliations:** Faculty of Metals Engineering and Industrial Computer Science, AGH University of Krakow, Al. Mickiewicza 30, Krakow, 30-059 Poland

Correction to: *Scientific Reports* 10.1038/s41598-025-88496-1, published online 13 February 2025

The original version of this Article contained an error in the title, where ‘Sn’ was incorrectly stated as ‘sn’. The title now reads:

**“Probing the local thermal expansion coefficient of single liquid Sn nanoparticles using EELS in STEM”**

Furthermore, in the original version of this Article an incorrect image was inadvertently published as Figure 2. The original Figure 2 and accompanying legend appear below.

**Fig. 2:**
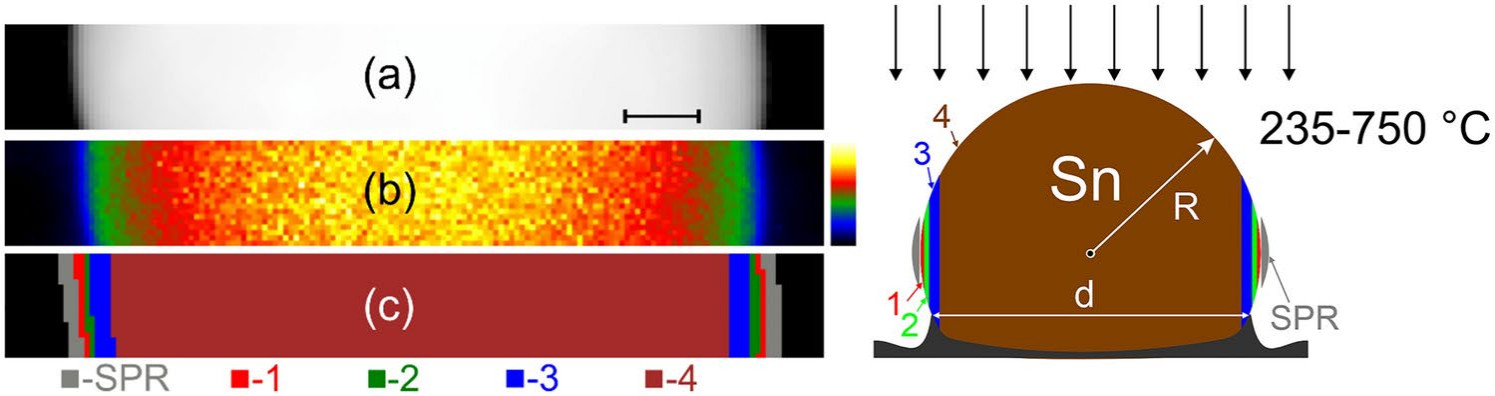
(**a**) HAADF STEM image of a selected part of the liquid Sn nanoparticle (yellow rectangle in Fig. 1a) at 700 °C, (**b**) false color EELS relative thickness map, and (**c**) false color map showing the regions “1”-“4” and “SPR” that were analyzed. (**d**) Schematic side-view of the Sn nanoparticle, indicating the approximate location of the analyzed regions. *R* and *H* represent the radius and height of the particle, respectively, and *d* represents the diameter of the contact area with the substrate. The scale bar is 20 nm in (a), and 0.1–1.6 t/λ in (b).

The original Article has been corrected.

