# Supplementary material for: Probing the local thermal expansion coefficient of single liquid Sn nanoparticles using EELS in STEM
Source: Sci Rep. 2025 Feb 13;15:5335. doi: 10.1038/s41598-025-88496-1 (PMC11825735; doi:10.1038/s41598-025-88496-1)
Supplement: Supplementary file 1 — Supplementary Material 1. Thermal stability of island Sn films on Si3N4 substrate. Deformation of the substrate under Sn nanoparticles. Figure S4. The measured volume plasmon Sn in a set of repeated EELS measurements. Results of measurements for other Sn nanoparticles. (PDF) [file 41598_2025_88496_MOESM1_ESM.pdf]

Supplementary Material for

Probing the Local Thermal Expansion Coefficient in Single Liquid Sn Nanoparticles Using EELS in STEM

A. Kryshtal, O. Khshanovska

Faculty of Metals Engineering and Industrial Computer Science, AGH University of Krakow, Al. Mickiewicza 30, Krakow 30-059, Poland

I. Thermal Stability of Island Sn Films on Si_3N_4 Substrate

1. Thermodynamic analysis

The temperature dependence of the vapor pressure of materials is commonly expressed by the Clausius-Clapeyron equation. However, a more practical expression for this relationship is given by:

$$\log P(atm) = A + B \cdot T^{-1} + C\ \log T + D \cdot T^{-3}. \tag{1}$$

The constants A, B, C, and D for liquid Sn are 5.262, -15332, 0, and 0, respectively.[1]

The vapor pressure of nanoparticles, however, is higher than that of a flat surface. For a spherical nanoparticle with radius *r*, the vapor pressure *P*(*r*) is described by the Kelvin equation[2]:

$$P(T,r) = P_{\infty}(T)\exp\left(\frac{2\mathrm{V_m}}{RT}\frac{\sigma}{r}\right), \tag{2}$$

where $P_{\infty}(T)$ is the bulk vapor pressure, σ is the surface tension, V_m is the molar volume, R is the universal gas constant.

Under perfect vacuum conditions, the maximum evaporation molar flux, m_{max}, is given by the Hertz-Knudsen equation[3]:

$$m_{max} = \frac{\alpha P(T)}{\sqrt{2\pi MRT}}, \tag{3}$$

where α is the evaporation coefficient (typically between 0 and 1, used to match experimental results to theoretical predictions), and M is the molecular weight of the material.

When the vacuum is not perfect, the net evaporation flux is expressed as

$$m = \frac{\alpha(P(T) - P_{amb})}{\sqrt{2\pi MRT}}, \tag{4}$$

where P_{amb} – is the ambient gas pressure. Importantly, the evaporation ceases when $P(T)>P_{amb}$, due to the establishment of dynamic equilibrium between evaporation and condensation.

The vapor pressure of liquid Sn, according to Equation (1), is approximately 10^{-8} and 10^{-7} Torr at 700 °C and 750 °C, respectively. These values are comparable to the typical base pressure of a TEM. Therefore, no evaporation of bulk Sn is expected at temperatures up to approximately 700 °C. At higher temperatures, evaporation may occur, with the rate depending on the actual pressure in the specimen area and the size of the nanoparticles. The radius of a Sn nanoparticle should be approximately 20 nm to exhibit a 10% increase, and 3 nm to exhibit a 100% increase in vapor pressure at 750 °C, as predicted by the Kelvin equation (2). Consequently, nanoparticles with sizes of several dozen nanometers should remain relatively stable at a temperature of 750 °C in a vacuum of 10^{-7} Torr. In contrast, particles smaller than 10–20 nm will have a noticeably higher vapor pressure compared to the bulk and are likely to evaporate at 750 °C.

2. *Experimental study*

To investigate the thermal stability of Sn nanoparticles at high temperatures, we used a 40 nm-thick Sn film deposited on a Si_3N_4 membrane of a Wildfire$_{HB}$ GT nano-chip (DENSsolutions). The study was conducted using a Tecnai G2 microscope operated at 200 kV over a temperature range of 600–900 °C. The base vacuum in the specimen chamber of the microscope was about 8×10^{-8} Torr. TEM images were analyzed using the ImageJ program.[4]

The as-deposited Sn film was annealed at 750 °C for less than a minute to form nanoparticles. A BF TEM image of the film is shown in Fig. S1a. The film contained well-separated liquid Sn particles with sizes ranging from 15 to 850 nm. As the vapor pressure of nanoparticles depends on their size (eq. 2), the presence of fine particles alongside larger ones in Fig. S1a indicates that no substantial evaporation of Sn occurred during the short-term annealing at 750 °C. Nevertheless, we cannot exclude the evaporation of nanoparticles smaller than approximately 15 nm.

The sample was sequentially annealed at 700 °C for 32 minutes (Fig. S1b), 750 °C for 17 minutes (Fig. S1c), 800 °C for 8 minutes (Fig. S1d), 850 °C for 2 minutes (Fig. S1e), and 900 °C for 5 minutes (Fig. S1f). During each annealing step, the specimen holder was retracted from the pole piece of the microscope to prevent possible contamination, and the images were acquired at a safe temperature of 600 °C. The number of particles in the images and the mean nanoparticle size as a function of annealing time and temperature are presented in Fig. S2a.

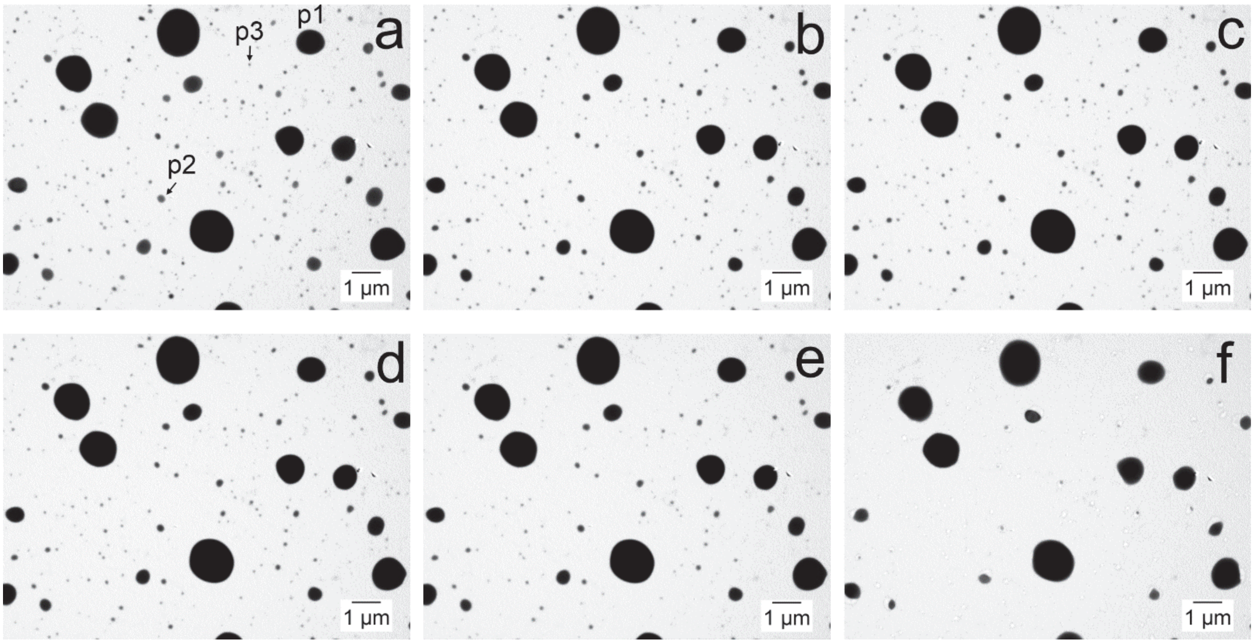

Figure S1. Bright-field TEM images of the same region of Sn film on a Si_3N_4 substrate annealed at different temperatures: (a) initial state, (b) 700 °C, (c) 750 °C, (d) 800 °C, (e) 850 °C, and (f) 900 °C. p1-p3 indicate nanoparticles selected for detailed analysis.

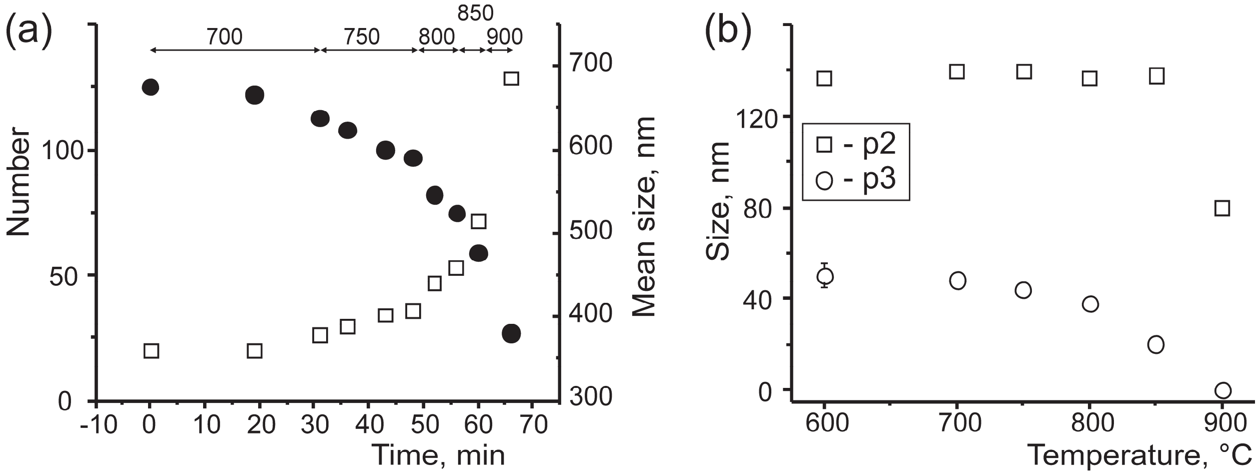

Figure S2. (a) The number of particles and the average nanoparticle size as functions of annealing time and temperature. (b) Projected area diameters of nanoparticles p1–p3 (Fig. S1) at different temperatures.

Our observations showed that Sn nanoparticles were stable at 700 °C (Fig. S1b) and relatively stable at 750 °C (Fig. S1c). The number of particles (*N)* in the TEM images remained constant during the first 21 minutes of annealing at 700 °C, followed by a 10% decrease in *N* and a simultaneous 5% increase in the mean particle size during the subsequent 11 minutes of annealing (Fig. S2a). Annealing at 750 °C for 16 minutes resulted in a further 10% decrease in the particle count due to the evaporation of the smallest nanoparticles (Fig. S1c, Fig. S2a).

A substantial decrease in the particle count was observed after several minutes of annealing at 800 °C and 850 °C (Fig. S2a), indicating an intensification of the evaporation process. Annealing at 900 °C drastically altered nanoparticles, resulting in the complete disappearance of nanoparticles smaller than approximately 70 nm (Fig. S1f) and a significant reduction in the size of the remaining particles (Fig. S2a).

To quantify the onset temperature of evaporation, we manually measured the size of three selected nanoparticles (*p1*, *p2*, and *p3* in Fig. S1a) as a function of annealing temperature. The initial sizes of nanoparticles *p1*, *p2*, and *p3* were 514 nm, 137 nm, and 50 nm, respectively. The

results for nanoparticles *p2* and *p3* are shown in Fig. S2b. Nanoparticle *p1* exhibited behavior similar to *p2* and is therefore not included in the graph.

The size of *p2* remained unchanged in the temperature range of 700–850 °C, decreasing to 80 nm only after annealing at 900 °C (Fig. S2b). In contrast, nanoparticle *p3* was stable at 700 °C, with a slight size reduction to 44±5 nm observed at 750°C, followed by a drop to 20 nm at 850 °C and complete disappearance at 900 °C (Fig. S2b).

As a result, we found that the onset temperature of evaporation in a vacuum of approximately 10^{-7} Torr was 750 °C for nanoparticles with a size of about 50 nm and 850 °C for nanoparticles larger than approximately 137 nm. Great care must be taken when heating fine Sn nanoparticles in a TEM to temperatures above 700 °C to avoid their evaporation. Conversely, several minutes of annealing a Sn film at 750 °C will result in the formation of a relatively stable array of Sn nanoparticles with sizes above 50 nm.

II. Deformation of the Substrate Under Sn Nanoparticles

Partial evaporation of Sn particles annealed at 900 °C exposed the underlying Si_3N_4 substrate, enabling its examination. For this part of the study, we used a probe Cs-corrected Titan Cubed G2 60-300 (FEI) TEM, employing the techniques described in the main text of the manuscript. The microscope's acceleration voltage was reduced to 80 kV to decrease the electrons' mean free path.

Figure S3a shows a HAADF STEM image of a partly evaporated Sn nanoparticle at room temperature. Deformation of the thin Si_3N_4 substrate beneath the particle and the formation of a "wetting ridge" at the droplet rim (indicated by the arrow in Fig. S3a) are visible. To quantify the deformation profile, we obtained an electron energy loss spectroscopy relative thickness map of this region (Fig. S3b). Figure 6c presents the substrate thickness profile along the dotted line in Figure S3b, calculated using an electron mean free path in Si_3N_4 of 84 nm. The height of the wetting ridge formed by the Sn droplet resting on the thin Si_3N_4 substrate was approximately 10 nm.

We believe that the ridge formed during the first temperature cycling of the particles. The as-deposited Sn film was continuous, and the thin substrate was likely deformed during the dewetting of the Sn film and the formation of nanoparticles.

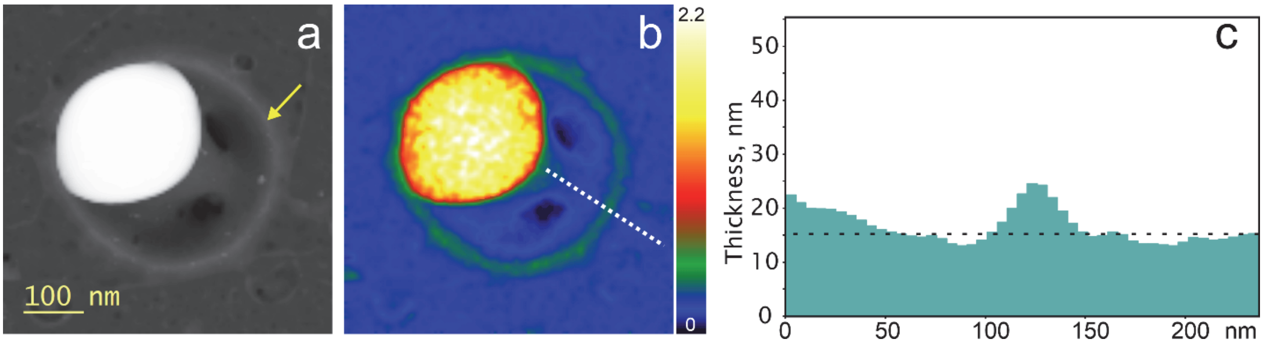

Figure S3. (a) HAADF-STEM image and (b) false-color relative thickness (t/λ) map of a partly evaporated Sn nanoparticle on a thin Si_3N_4 substrate. (c) Substrate thickness profile along the dotted line in (b). The dashed line in (c) indicate the level of a flat substrate.

III. Images and Graphs for the Main Text

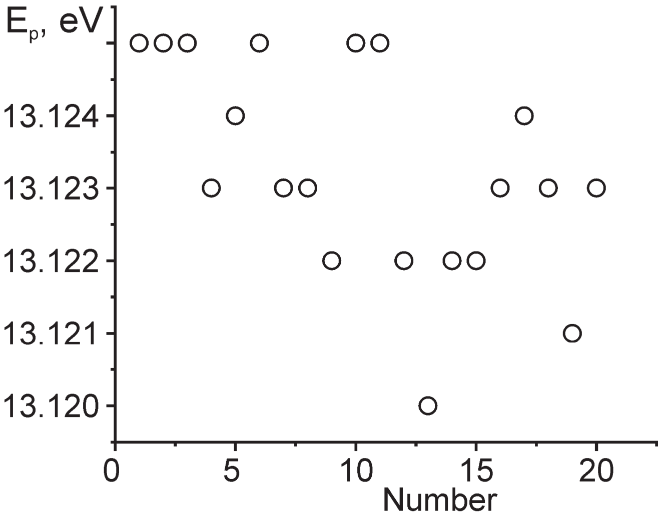

Figure S4. The measured volume plasmon energy E_p of Sn in a set of repeated EELS measurements from different regions of the nanoparticle.

IV. Results of Measurements for Other Sn Nanoparticles

EELS maps were collected for four individual liquid nanoparticles with sizes of 315 nm, 190 nm, 53 nm, and 41 nm. The data for the 190 nm particle are presented in the main text, while the measurement results for the other Sn nanoparticles are summarized in Table S1.

These data support and validate the conclusions drawn in the main text. For example, the CTE values in the core of the nanoparticles varied from 5.3 to 5.86x10^{-5} °C^{-1}, which is significantly lower than the handbook value of 9-10x10^{-5} °C^{-1}. A slightly smaller CTE value for the 41 nm and 53 nm nanoparticles suggests a stronger substrate effect on their CTE.

A CTE gradient along the radius was observed in each nanoparticle studied. However, the magnitude of this effect varied from particle to particle. For instance, the gradient in the 315 nm nanoparticle was consistent with that seen in the 190 nm nanoparticle, where the CTE value at the free surface approached the reference value for liquid Sn. In contrast, the 41 nm and 53 nm nanoparticles displayed a smaller CTE gradient along their radii, likely due to stronger interactions with the Si_3N_4 substrate.

Thus, the contact angles of 41 nm and 53 nm nanoparticles, assessed under the assumption of a flat and rigid substrate using Eq. 2 of the main text, were determined to be 91° and 112°, respectively, at a temperature of 250 °C. These angles are smaller than those observed for larger nanoparticles. As a result, in the EELS maps, representing a two-dimensional projection of the sample, only a very small region of the nanoparticle remains out of contact with the substrate, complicating any analysis of the particle's free surface. Consequently, the smaller CTE gradient observed in these particles can be attributed to the substrate effect, which is consistent with the conclusions drawn in the main text.

Table S1. Temperature shift of the plasmon energy (dE/dT) and local coefficient of volumetric thermal expansion (α_V) of liquid Sn nanoparticles of sizes 315 nm, 53 nm, and 41 nm on a Si_3N_4 substrate. Here, *d* represents the approximate inward distance from the surface of the Sn nanoparticle.

d, nm	dE/dT, [meV/°C]	α_V, 10^{-5} [°C^{-1}]
315 nm		
0-1.5	-0.7±0.09	10.7
1.5-3	-0.44±0.03	6.8
3-9	-0.382±0.005	5.9
> 9	-0.381±0.005	5.9
53 nm		
0-2.5	-0.48±0.04	7.3
2.5-5	-0.398±0.02	5.8
5-10	-0.366±0.008	5.5
> 10	-0.353±0.004	5.3
41 nm		
0-2	-0.387±0.01	5.9
2-4	-0.32±0.01	4.9
4-7	-0.351±0.005	5.4
> 7	-0.362±0.005	5.6

REFERENCES

1. Atkins, P., Paula, J. de & Keeler, J. *Atkins' Physical Chemistry. Oxford University Press* (Oxford University Press, 2022). doi:10.1093/hesc/9780198847816.001.0001.
2. Thomson, W. 4. On the Equilibrium of Vapour at a Curved Surface of Liquid. *Proceedings of the Royal Society of Edinburgh* **7**, 63–68 (1872). doi:10.1017/S0370164600041729
3. Kolasinski, K. W. *Surface Science: Foundations of Catalysis and Nanoscience*. *Surface Science: Foundations of Catalysis and Nanoscience: Third Edition* (2012). doi:10.1002/9781119941798.
4. Schneider, C. A., Rasband, W. S. & Eliceiri, K. W. NIH Image to ImageJ: 25 years of image analysis. *Nature Methods* vol. 9, 671–675, (2012) doi:10.1038/nmeth.2089.
